# Enhancing immunogenicity and transmission-blocking activity of malaria vaccines by fusing Pfs25 to IMX313 multimerization technology

**DOI:** 10.1038/srep18848

**Published:** 2016-01-08

**Authors:** Yuanyuan Li, Darren B. Leneghan, Kazutoyo Miura, Daria Nikolaeva, Iona J. Brian, Matthew D. J. Dicks, Alex J. Fyfe, Sarah E. Zakutansky, Simone de Cassan, Carole A. Long, Simon J. Draper, Adrian V. S. Hill, Fergal Hill, Sumi Biswas

**Affiliations:** 1 Jenner Institute, University of Oxford, Oxford, OX3 7DQ, UK; 2 Laboratory of Malaria and Vector Research, National Institute of Allergy and Infectious. Disease/National Institutes of Health, Rockville, Maryland, USA; 3 IMAXIO, 69007 Lyon, France

## Abstract

Transmission-blocking vaccines (TBV) target the sexual-stages of the malaria parasite in the mosquito midgut and are widely considered to be an essential tool for malaria elimination. High-titer functional antibodies are required against target antigens to achieve effective transmission-blocking activity. We have fused Pfs25, the leading malaria TBV candidate antigen to IMX313, a molecular adjuvant and expressed it both in ChAd63 and MVA viral vectors and as a secreted protein-nanoparticle. Pfs25-IMX313 expressed from viral vectors or as a protein-nanoparticle is significantly more immunogenic and gives significantly better transmission-reducing activity than monomeric Pfs25. In addition, we demonstrate that the Pfs25-IMX313 protein-nanoparticle leads to a qualitatively improved antibody response in comparison to soluble Pfs25, as well as to significantly higher germinal centre (GC) responses. These results demonstrate that antigen multimerization using IMX313 is a very promising strategy to enhance antibody responses against Pfs25, and that Pfs25-IMX313 is a highly promising TBV candidate vaccine.

Despite recent efforts, malaria still remains a major public health problem that affects nearly 50% of the world’s population. In 2013 approximately 3.3 billion people were at risk of contracting malaria and there were an estimated 198 million cases resulting in approximately 584,000 deaths according to World Malaria Report^1^. *Plasmodium falciparum* (*P. falciparum*) is responsible for most of the morbidity and mortality attributed to malaria, and has been the main focus of the malaria vaccine development field^2^. Most *P. falciparum* vaccines are designed to target a specific parasite stage. Vaccines targeting the pre-erythrocytic and erythrocytic stages of malaria have received great attention as they can provide protection against infection and clinical disease. The most advanced malaria vaccine is RTS,S (pre-erythrocytic malaria vaccine), which has recently completed a Phase III clinical trial, has a relatively short-lived efficacy of 46% against clinical malaria and 34% against severe malaria in children and older infants, and the efficacy is lower in younger infants^3^. While this is a promising start and a milestone for the field, malaria elimination will only come with a more effective second-generation vaccine which could be used either alone or in combination with RTS,S. The updated 2030 Strategic Goal of the Malaria Vaccine Technology Roadmap now calls for development of vaccines which reduce transmission, thereby substantially reducing incidence and enabling elimination in multiple settings^4^. TBVs aim to induce high-titer functional antibodies against target antigens and mediate protective efficacy by neutralizing sexual-stage parasite development in the mosquito host. Vaccines against the pre-erythrocytic and erythrocytic stage of the parasite may also play a role in reducing transmission.

The most clinically advanced TBV candidate antigen is Pfs25, a 25 kDa protein, expressed on the surface of zygotes and ookinetes in the mosquito midgut^5^. Other well studied TBV antigen candidates include Pfs230 and Pfs48/45^6^. Anti-Pfs25 antibodies induced by a range of different formulations (a comprehensive list of which has recently been reviewed by Nikolaeva *et al.*^7^) have been shown to achieve high transmission-blocking activity (TBA) (inhibition of parasite prevalence in mosquito) and TRA (inhibition of parasite infection intensity in mosquito) in pre-clinical studies.

One of the major challenges in translating pre-clinical efficacy of TBVs to humans has been the apparent need for exceptionally high antibody titers against Pfs25 to achieve effective TBA. A Phase Ia clinical trial of Pfs25 protein formulated in Montanide ISA51 adjuvant showed that the anti-Pfs25 antibodies induced after vaccination can indeed block transmission of the parasite in both the SMFA^8^ and direct membrane feeding assay (DMFA)^9^, but this activity required high antibody titers. This trial was, however, halted due to safety concerns related to the adjuvant. In order to improve the immunogenicity of Pfs25-based vaccines, several methods are being investigated. Pfs25 fused to a detoxified form of *Pseudomonas aeruginosa* exoprotein A (EPA), forming a nanoparticle with hydrodynamic radius ranging in size from 5 to 25 nm^10^, has been shown to increase antibody responses versus unconjugated Pfs25 between 75- and 100-fold^11^. A batch of Pfs25-EPA has been manufactured according to Good Manufacturing Practice (GMP) and has entered Phase Ia/b clinical trials (ClinicalTrials.gov Identifier: NCT01434381 and NCT01867463). Similarly, Pfs25 has been fused to the outer-membrane protein complex (OMPC) of *Neisseria meningitidis* serogroup B. This Pfs25-OMPC induced a substantial increase in anti-Pfs25 antibodies in mice compared to a similar dose of Pfs25 alone, as well as demonstrating a response sustained for over 18 months in rhesus monkeys^12^. A virus-like particle (VLP) has been engineered to display Pfs25 on its surface; the coat protein (CP) of Alfalfa mosaic virus was fused to Pfs25 and expressed in *Nicotiana benthamiana*. Antibodies produced by mice immunized with the Pfs25-CP VLP had complete TBA throughout the 6 months of the study period^13^. These formulations are not without their downsides however: conjugation of Pfs25 to EPA or OMPC requires production of each from a different system (*P. pastoris, Escherichia coli* and *N. meningitidis*) followed by a chemical conjugation step. This adds time, expense and it can be difficult to reproducibly repeat conjugations with similar efficiency; while manufacture of Pfs25-VLP in *N. benthamiana* requires a lot of time to grow sufficient plant biomass to purify large amounts of vaccine.

In 2007, Kubler-Kielb *et al.* showed that conjugation of Pfs25 to itself significantly improved its immunogenicity^14^. Here, we have used a novel technology called IMX313, based on a chimeric version of the oligomerization domain from chicken complement inhibitor C4b-binding protein (C4 bp)^15^, in order to obtain homogenous, self-assembling oligomers of Pfs25. This C4 bp oligomerization domain has been shown to spontaneously form soluble heptameric structures (termed nanoparticles in this study) when expressed in *E. coli*, and protein antigens fused to these domains have been shown to improve antibody responses over the same amount of monomeric antigen^15^. In addition, mice immunized with the blood-stage malaria vaccine candidate antigen MSP1_19_ fused to IMX313 (expressed in *E. coli*) were protected against challenge with a lethal dose of *Plasmodium yoelii* parasites^15^. Other studies have demonstrated that fusion of an antigen to IMX313 has a number of beneficial adjuvant effects. Immunization of mice with the *Mycobacterium tuberculosis* antigen 85A fused to IMX313 in both DNA vaccines and viral vectors showed consistent increases in CD4^+^ and CD8^+^ T cell responses. This same fusion induced higher IFN-γ responses in rhesus macaques and improved the quantity of the immune response in both mice and monkeys without changing the quality^16^. A viral vector vaccine encoding 85A-IMX313 has now entered Phase I clinical trial in healthy UK adults (NCT01879163).

In this study, we investigated the potential of using the IMX313 multimerization strategy to improve the immunogenicity and transmission-blocking efficacy of vaccines targeting Pfs25. We have fused Pfs25 to IMX313 and expressed it from the leading clinical viral vectors, chimpanzee adenovirus serotype 63 (ChAd63) and modified vaccinia virus Ankara (MVA)^17^. Notably these viral vectors (ChAd63-MVA) expressing Pfs25 have been previously reported in a pre-clinical study to induce antibodies that exhibit functional TBA and TRA in the SMFA^18^. We have also produced Pfs25-IMX313 as a secreted protein-nanoparticle in *P. pastoris*. Both the viral vectored Pfs25-IMX313 and *P. pastoris* expressed protein-nanoparticle (formulated in Alhydrogel) showed significantly improved antibody responses in mice and subsequent TRA in SMFA compared to using monomeric Pfs25 alone in both vaccination regimes. We also assessed the quality of the immune response to Pfs25-IMX313 compared to Pfs25 alone, and assessed the cellular mechanisms associated with the increased antibody response.

## Results

### Fusion to IMX313 heptamerizes Pfs25

**Figure 1:**
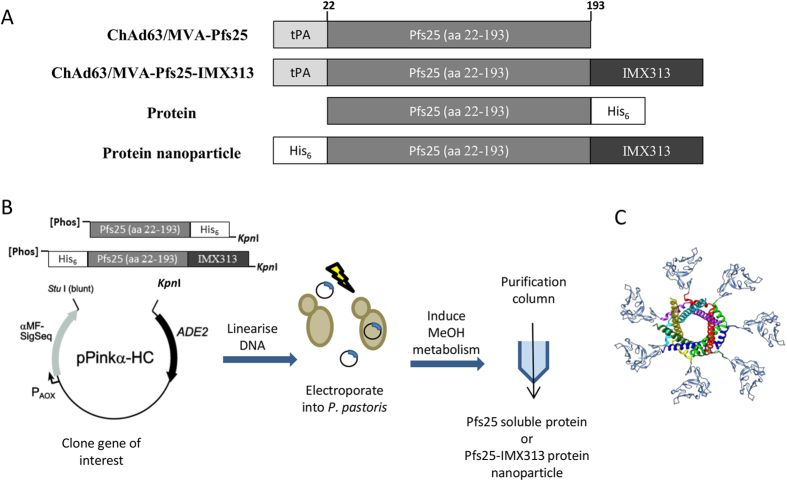
Schematic representation of the construction and production of Pfs25 and Pfs25-IMX313 in viral vectors and *P. pastoris*. (**A**) Four constructs used in these experiments: **in viral vectors** 1) Pfs25 (aa 22–194) fused to an N-terminal secretion signal peptide tPA; 2) Pfs25-IMX313 fused to tPA; **and in** ***P. pastoris*** 3) Pfs25 (aa 22–194) with a C-terminal hexahistidine (His6) tag; 4) Pfs25 fused to IMX313 with an N-terminal His6 tag. (**B**) Schematics showing the steps in production of Pfs25 and Pfs25-IMX313 in *P. pastoris*. The antigen sequences were cloned into the *P. pastoris* expression plasmid pPinkα-HC (Invitrogen^TM^, Life Technologies, UK) (containing the α-mating factor secretion signal peptide). After electroporation of target plasmids into *P. pastoris*, positive colonies were screened for protein expression and successful candidates were selected for protein production as indicated. (**C**) Idealised structure of the Pfs25-IMX313 heptamer. Pfs25 (blue) homology model based on the crystal structure of Pvs25 (PDB: 1Z27) fused N-terminally to IMX313 represented here by the crystal structure of the human C4bp (PDB: 4B0F).

**Figure 2:**
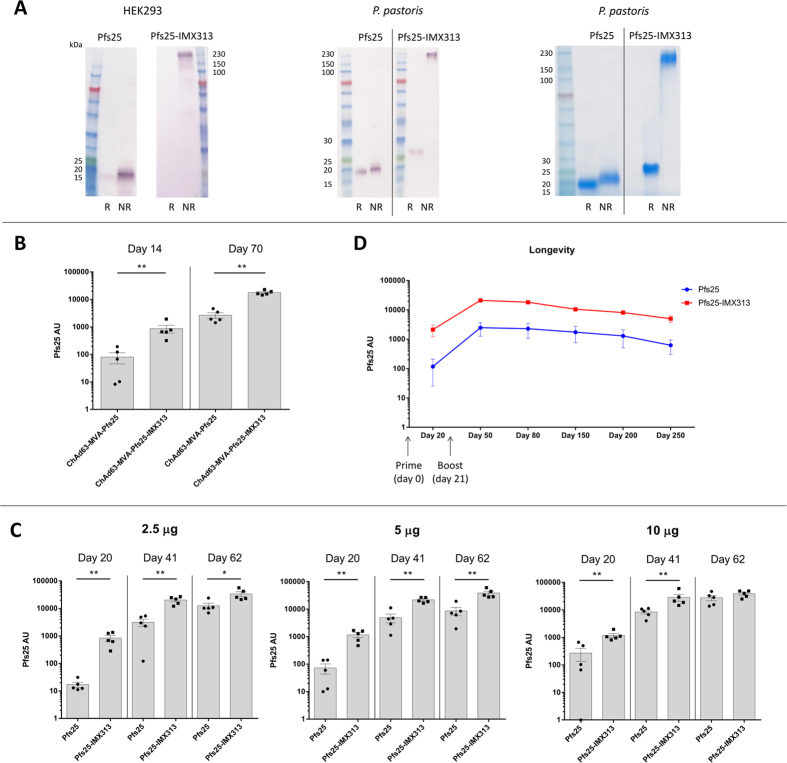
Expression of Pfs25, Pfs25-IMX313 and antigen-specific IgG responses in mice after immunization with viral-vectors and protein-in-adjuvant formulations. (**A**) Western blot of supernatant from pENTR4-LPTOS shuttle plasmids transfected into HEK293 cells (left panel) and protein purified from *P. pastoris* supernatant (middle panel) for Pfs25 and Pfs25-IMX313 under reduced (R) and non-reduced (NR) conditions. Western blotting was performed using anti-Pfs25 4B7 mAb. The purified protein was also analyzed on SDS-PAGE using coomassie stain (right panel). For the western blots, 15 μL supernatant from transfected HEK293 cells and 500 ng of purified proteins from *P. pastoris* were loaded per lane. For Coomassie analysis, 5 μg of purified proteins were loaded. (**B**) Two groups (n = 5 per group) of BALB/c mice were primed with 1X10^8^ i.u of ChAd63 (expressing Pfs25 or Pfs25-IMX313) on day 0 and boosted 8 weeks later with 1X10^7^ pfu of MVA (expressing Pfs25 or Pfs25-IMX313) via the intramuscular (i.m.) route. Day 14 and day 70 serum samples were collected and the total IgG response against Pfs25 was measures using standardized ELISA. (**C**) Six groups (n = 5 per group) of BALB/c mice were immunized i.m. in parallel with 2.5 μg, 5 μg and 10 μg of soluble Pfs25 or Pfs25-IMX313 nanoparticle formulated in Alhydrogel. Mice were primed on day 0 and boosted on day 21 followed by another boost on day 42. Day 20, 41 and 62 serum were collected and analyzed by Pfs25 standardized ELISA. (**D**) Two groups (n = 5 per group) of BALB/c mice were immunized via the i.m. route with 2.5 μg of Pfs25 or Pfs25-IMX313 formulated in Alhydrogel. Mice were primed on day 0 and boosted on day 21. Serum was collected on days 20, 50, 80, 150, 200 and 250 and anti-Pfs25 total IgG was measured. Mean with SEM are depicted for (**B–D**). Individual data are shown for B and C. Mann-Whitney test was performed *p < 0.05, **p < 0.01.

Pfs25 (GeneBank locus AAN35500, from Alanine (Ala)-22 to Threonine (Thr)-193) with three potential N-linked glycosylation sites (112, 165 and 187) mutated, as described previously^19^, as well as the Pfs25-IMX313 fusion construct, were expressed either *in situ* using the viral-vector ChAd63-MVA regime described in the materials and methods (Fig. 1A) or as secreted proteins in *P. pastoris* (Fig. 1B). Insertion of Pfs25-IMX313 into the *P. pastoris* genome was done by homologous recombination of one or more copies of the expression plasmid into the TRP2 gene. The gene for the Pfs25-IMX313 protein-nanoparticle was inserted under the control of an alcohol oxidase 1 (AOX1) promoter allowing methanol-inducible protein expression. We modelled the imagined heptamer structure of Pfs25-IMX313 (Fig. 1C) based on the existing crystal structures of Pvs25 (PDB: 1Z27^20^) and the human C4bp (PDB: 4B0F^21^). To test the *in vitro* expression of the antigens (as would occur from the viral-vector vaccines), pENTR4-LPTOS shuttle plasmid DNA^22^ expressing Pfs25 or Pfs25-IMX313 (both under the control of the human cytomegalovirus (CMV) promoter^23^) was used to transfect HEK293 cells. The supernatant was collected and analyzed by western blot using the 4B7 monoclonal antibody (mAb). Pfs25 protein and Pfs25-IMX313 protein-nanoparticle purified from *P. pastoris* were similarly analyzed using western blot. The western blots were performed under both reducing and non-reducing SDS-PAGE conditions (Fig. 2A left panel and middle panel). The HEK293 cell and *P. pastoris* expressed proteins migrated at the predicted molecular weight of Pfs25 (19.62 kDa) and Pfs25-IMX313 (25.92 kDa) under reducing conditions, although the 4B7 mAb was unable to detect Pfs25-IMX313 expressed by HEK293 cells under reducing conditions. The 4B7 mAb is known to be partially conformation dependent, binding strongly to non-reduced Pfs25 and only weakly to the reduced antigen^24^. As expected 4B7 recognizes the non-reduced form of the protein at the strongest intensity for both the *P. pastoris* and HEK293 cell produced proteins. Under non-reducing conditions, both Pfs25 and Pfs25-IMX313 were recognised by 4B7 and the band detected for Pfs25-IMX313 was <230 kDa and >150 kDa. These data suggest the presence of disulphide-linked homogeneous multimeric protein which is assumed to be heptameric (7*27 kDa = 189 kDa), based on its high molecular weight as well as previous reports^15^. Analysis by Coomassie stained SDS-PAGE of the Pfs25 protein and Pfs25-IMX313 protein-nanoparticle purified by nickel-chelate affinity chromatography and size exclusion chromatography (SEC) (Fig. 2A right panel) demonstrated that the purified proteins were homogenous and pure.

### Immunogenicity of Pfs25-IMX313 expressed from viral-vectors or as a protein-nanoparticle

BALB/c mice were primed (day 0) with 1 × 10^8^ infectious units (i.u.) of either ChAd63-Pfs25 or ChAd63-Pfs25-IMX313 and boosted 8 weeks later (day 56) with 1 × 10^7^ plaque forming units (pfu) of MVA-Pfs25 or MVA-Pfs25-IMX313 respectively. Sera were collected from the mice on days 14 (2 weeks post-prime) and 70 (2 weeks post-boost) and anti-Pfs25 total IgG responses were measured in the serum using a standardized ELISA^25^. At both time-points tested, the ChAd63_MVA Pfs25-IMX313 regime produced significantly higher serum anti-Pfs25 IgG responses compared to the ChAd63_MVA Pfs25 regime (Fig. 2B). Notably, the level of antibodies induced after priming alone with ChAd63-Pfs25-IMX313 was almost comparable to that induced after prime-boost vaccination with the ChAd63_MVA expressing Pfs25.

Similarly, Pfs25-IMX313 protein-nanoparticle produced in *P. pastoris* induced significantly higher levels of anti-Pfs25 IgG in BALB/c mice than soluble Pfs25 protein. Mice were vaccinated with 2.5, 5 or 10 μg of Pfs25 or Pfs25-IMX313 formulated in Alhydrogel at days 0, 21 and 42 and the antibody response was measured 3 weeks after each vaccination on days 20, 41 and 62. At every dose and time-point tested, the Pfs25-IMX313 protein-nanoparticle induced significantly higher anti-Pfs25 antibodies than monomeric Pfs25 (Fig. 2C), except with a 10 μg dose on day 62 which may correspond to a plateau in inducible-antibody levels in mice. Furthermore, two doses of 2.5 μg Pfs25-IMX313 (Fig. 2C, Day 41) was as immunogenic as three doses of monomeric Pfs25 at 10 μg (Fig. 2C, Day 62). Vaccination with monomeric Pfs25 resulted in a dose-dependent induction of anti-Pfs25 IgG (p = 0.017), however there was no dose-effect in Pfs25-IMX313 groups (p = 0.326) which suggests there might be a dose-sparing effect (as calculated by linear regression analysis using Log_10_ transformed antibody responses for each vaccine group (Pfs25 or Pfs25-IMX313)).

In addition, the serum IgG antibody response in mice after vaccination with soluble Pfs25 or Pfs25-IMX313 nanoparticle formulated in Alhydrogel (prime on day 0 and boost on day 21) was monitored over 250 days (Fig. 2D). Pfs25-IMX313 induced anti-Pfs25 IgG levels that decreased at a similar rate after boosting, although the anti-Pfs25-IMX313 response was consistently higher than that of Pfs25 throughout.

### Pfs25-IMX313 vaccination did not induce auto-reactive antibodies

IMX313 is a hybrid of the two chicken homologues of the oligomerization domain of human C4bp (HC4bp), with 21% homology (11 identical residues in an overlap of 52 amino acids) to the sequence of the human protein^15^ (Fig. S1A). It is thus unlikely that vaccination with Pfs25-IMX313 would generate an immune response which cross-reacts with HC4bp. In order to confirm this we tested the serum from vaccinated mice for reactivity to both IMX313 and HC4bp in an ELISA. We investigated the ability of day 70 sera from Fig. 2B and day 62 sera from Fig. 2C (2.5 μg dose) to bind to purified recombinant IMX313 protein, and HC4bp (purified from plasma). Anti-IMX313 IgG was detected in the serum of mice from the Pfs25-IMX313 vaccinated group (Fig. S1B), but this same sera did not recognize HC4bp (Fig. S1C). Sera from mice vaccinated with HC4bp were used as a positive control.

### Vaccine-induced anti-Pfs25 antibodies recognize native parasite protein

**Figure 3:**
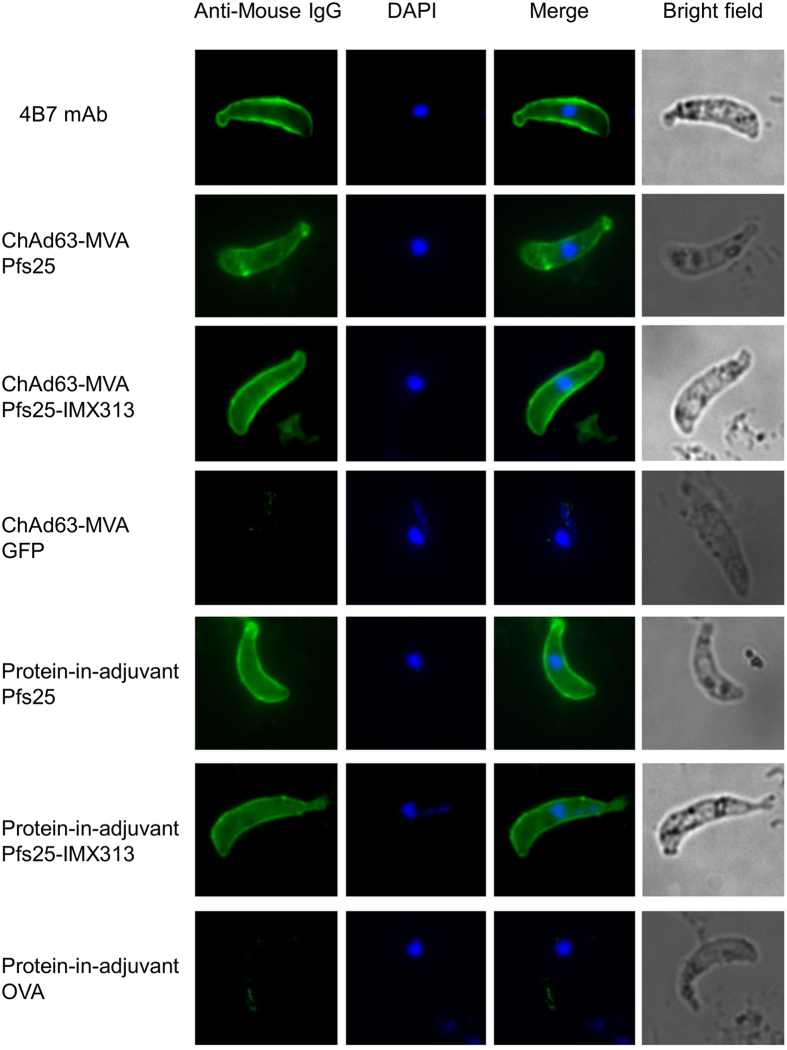
Reactivity of vaccine-induced antibodies to native parasite antigen. Ookinetes of *P. berghei* transgenic for Pfs25 (Pfs25DR3) were stained with pooled sera from mice that received ChAd63-MVA expressing Pfs25 or Pfs25-IMX313 (Fig. 2B), and mice that received 3 doses of 2.5 μg Pfs25 or Pfs25-IMX313 formulated in Alhydrogel (Fig. 2C). Anti-Pfs25 mAb 4B7 was included as the positive control and sera from mice which received ChAd63-MVA expressing GFP or three doses of OVA protein formulated in Alhydrogel were included as negative controls. Antibody binding was detected by Alexa Fluor® 488-conjugated goat anti-mouse IgG (green) (lane 1) and the DNA was stained with DAPI (blue) (lane 2). Merged view (lane 3) as well as bright field (lane 4) are also shown.

Pfs25 is expressed on the surface of ookinetes in the mosquito midgut. We tested the ability of vaccine-induced antibodies (from Fig. 2B day 70 and Fig. 2C day 62 (2.5 μg)) to recognize Pfs25 expressed on the surface of the ookinetes of transgenic *P. berghei* parasites expressing Pfs25 (Pfs25DR3) by immunofluorescence. Pooled sera from mice, for both the viral-vector and protein-in-adjuvant immunized groups, recognized Pfs25 predominantly on the surface of the ookinete (Fig. 3). The staining pattern was identical to the positive control using the 4B7 mAb. Serum from vector-control (vectors expressing green fluorescent protein, GFP) and ovalbumin (OVA) protein immunized mice were used as negative controls for the viral-vector and protein-in-adjuvant groups respectively.

### Anti-Pfs25 antibodies block parasite-transmission

The functional activity of the anti-Pfs25 antibodies were tested using the SMFA. Serum was pooled from each group of vaccinated mice at the end of the vaccination schedule (2 weeks after prime-boost vaccination with viral-vectors (Fig. 2B) and 2 weeks after the third immunization with 2.5 μg protein-in-adjuvant vaccines (Fig. 2C)). The total IgG was purified using protein G columns and different concentrations of total IgG were mixed with *in vitro* cultured *P. falciparum* gametocytes and fed to *Anopheles stephensi* mosquitoes through a membrane feeder. Purified IgG from vector control and OVA immunized mice were used as negative controls for the viral-vector and protein-in-adjuvant studies respectively.

**Figure 4:**
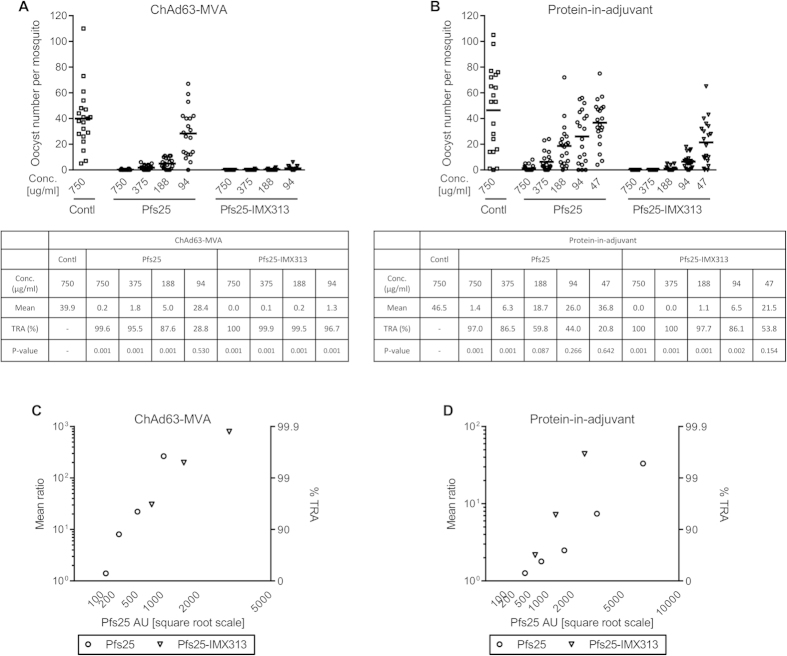
Effect of mouse IgG induced by vaccination on *P. falciparum* NF54 parasite infectivity in (A) *stephensi* mosquitoes. Total IgG was purified from pooled day 70 serum from mice vaccinated with ChAd63-MVA expressing Pfs25 or Pfs25-IMX313 (**A**), or day 62 pooled serum from mice that received three doses of 2.5 μg Pfs25 or Pfs25-IMX313 formulated in Alhydrogel (**B**). The purified IgG was mixed with *P. falciparum* NF54 cultured gametocytes and fed to *A. stephensi* mosquitoes (n = 20 per test group) in SMFA. Midguts were dissected 7 days post-feeding. Data points represent the number of oocysts in individual mosquitoes and the lines show the arithmetic mean. IgG from vector immunized mice and OVA protein immunized mice were used as negative control for (**A,B**) respectively. The tables show the mean number of oocysts per mosquito and percentage of transmission reducing activity with p-value. (**C**) The square root of anti-Pfs25 specific IgG level in the feeder is shown on the x-axis. The ratio of mean oocyst counts in control and test samples are plotted on a log scale along the left y-axis, and the associated % TRA is plotted along the right y-axis. The same data as (**C**) for the protein-in-adjuvant vaccination study is plotted in (**D**).

When anti-Pfs25 antibodies induced after vaccination with viral-vectors were tested at a concentration of 750 μg/ml of total IgG, both groups showed more than 99% TRA compared to the IgG from the vector control immunized group (Fig. 4A). For all other total IgG concentrations tested, IgG from the ChAd63_MVA Pfs25-IMX313 group had significantly fewer oocysts than the Pfs25 group (p < 0.001 for all). After the protein-in-adjuvant vaccinations with the monomeric Pfs25 and protein-nanoparticle Pfs25-IMX313, a similar effect was observed (Fig. 4B). At all concentrations, IgG from the Pfs25-IMX313 protein-nanoparticle vaccinated group had significantly better TRA activity than the monomeric Pfs25 group (p < 0.02 for all).

This difference in TRA could be solely due to more anti-Pfs25 IgG present in the sera from the Pfs25-IMX313 vaccinated groups, as demonstrated by the ELISA (Fig. 2B,C). Another possible contributing factor, which cannot be revealed by the standardized Pfs25 ELISA, is that expressing the antigen fused to IMX313 may have induced qualitatively different anti-Pfs25 IgG (e.g. recognition of different Pfs25 epitopes and/or better antibody avidity). To this end, anti-Pfs25 antibody levels in the purified IgGs (used for the SMFA) were determined by the standardized ELISA and re-analyzed with the SMFA data. When anti-Pfs25 antibody levels were adjusted, there was no significant group effect (i.e., either Pfs25 or Pfs25-IMX313) following ChAd63-MVA immunization (Fig. 4C, p = 0.359). However, in the protein-in-adjuvant study, there was a significant difference between the Pfs25 and Pfs25-IMX313 groups (Fig. 4D, p = 0.045). To confirm the difference in quality, the purified IgGs from the protein-in-adjuvant study were tested again by SMFA (Fig. S2). When data from the two feeding experiments were combined a higher significant difference in the SMFA-quality of antibodies was observed between groups (p = 0.009).

### Avidity and IgG subclass of vaccine-induced antibodies

**Figure 5:**
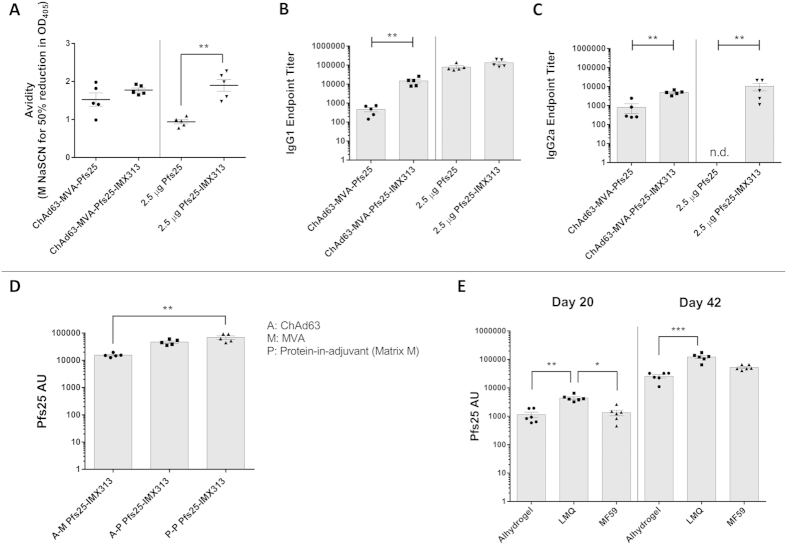
Quality difference in the antibody response after vaccination and comparison of Pfs25-IMX313 immunogenicity between different vaccination regimes and adjuvants. Antibody avidity was assessed in day 70 and day 62 serum from mice immunized with viral-vectors (Fig. 2B) and 2.5 μg protein-in-adjuvant vaccines respectively (Fig. 2C). Avidity of serum IgG responses was assessed by NaSCN-displacement ELISA and is reported as the molar concentration of NaSCN required to reduce the OD_405_ to 50% of that without NaSCN. The isotype profiles (IgG1 and IgG2a) of serum antibody responses were also assessed by ELISA for the same time-points for the viral-vector immunized mice (**B**) and the protein-in-adjuvant immunized mice (**C**) (n.d., not detected). BALB/c mice (n = 5 per group) received Pfs25-IMX313 vaccines via the i.m. route in different combination of regimes. A–M and A–P prime-boost regime were 8 weeks apart and P–P was 4 weeks apart. Two weeks after the boost vaccination in each group, serum samples were collected and the total IgG response was measured using a Pfs25 standardized ELISA (**D**). BALB/c mice (n = 6 per group) received prime-boost vaccinations (2 immunisations, 3 weeks apart) of Pfs25-IMX313 protein-nanoparticle formulated in Alhydrogel, LMQ or MF59. Three weeks after each vaccination, serum samples were collected and the total IgG response was measured using Pfs25 standardized ELISA (**E)**. Mean with SEM are depicted and individual data are shown for all figures. For A, B and C, Mann-Whitney test was performed **p < 0.01. For D and E, Kruskal-Wallis test followed by Dunn’s multiple comparison post-test was performed *p < 0.05, **p < 0.01, ***p < 0.001.

To further characterize the vaccine-induced antibody response, we investigated the avidity and IgG subclasses of antibodies induced after both viral-vector and protein-in-adjuvant vaccinations (day 70 after viral-vector vaccination and day 62 after protein-in-adjuvant for the 2.5 μg dose groups). Avidity ELISA was performed based on displacement by sodium thiocyanate (NaSCN). After protein vaccination, the avidity of the anti-Pfs25 antibodies induced by the Pfs25-IMX313 protein-nanoparticle was significantly higher than that of the antibodies induced by Pfs25 alone. However, after vaccination with viral vectors there was no significant difference in the avidity of the antibodies induced (Fig. 5A).

After viral-vector immunizations, Pfs25-IMX313 induced significantly higher IgG1 and IgG2a responses than the Pfs25 group (Fig. 5B). For the protein-in-adjuvant study, Pfs25-IMX313 induced a higher amount of IgG1 but this was not significantly different, whereas the IgG2a response was significantly higher in the Pfs25-IMX313 protein-nanoparticle vaccinated group (Fig. 5C). No IgG2a was detected in Pfs25 vaccinated mice, probably because Alhydrogel (alum-based adjuvant) is not a potent inducer of T-helper cell type 1 (Th1) response^26^. IgG1 and IgG2a have been used as markers of a T-helper cell type 2 (Th2) and Th1 response respectively^27^. Judging from the ratio of IgG1/IgG2a based on the ELISA data (Fig. 5B,C), it is clear that Pfs25 multimerization by IMX313 does not change the balance of Th2/Th1 responses if administrated in viral vectors and this regime generally induces a balanced Th2/Th1 response as reported in a previous study^18^. In contrast, compared to monomeric Pfs25, the Pfs25-IMX313 protein-nanoparticle was able to significantly boost the Th1 response in Alhydrogel and resulted in a more balanced Th2/Th1 profile.

### Comparison of vaccination regimes and adjuvants

We further investigated whether a combination of viral-vectored with protein-nanoparticle vaccines would lead to an increase in the immune response over either regime in isolation. Pfs25-IMX313 fusion vaccines were compared head to head as one of the following regimes; ChAd63-MVA (A–M), ChAd63-prime protein-in-adjuvant boost (A–P), and protein-in-adjuvant prime-boost (P–P), where the adjuvant was Matrix M. A priming vaccination with adenovirus induces an effective antibody response, and both A–M^18^,^28^ and A–P^29^ have been reported to induce strong T and B cell responses, whilst A–P has been shown to induce higher levels of antigen-specific IgG than three doses of protein vaccines formulated in several adjuvants tested^30^. In our study, sera from mice immunized by each of the three regimes were collected 2 weeks after the boost vaccination and we observed that only P–P regimes induced significantly higher antibody responses than A–M (Fig. 5D). This suggests that the P–P regime is the best way to induce antibody responses under the tested conditions of dosage and adjuvant.

We also investigated whether the immune response from P–P vaccination could be further improved through the use of other adjuvants. Mice were immunized with 2.5 μg Pfs25-IMX313 protein-nanoparticle formulated in Alhydrogel, oil-in-water emulsion MF59 (Novartis) or LMQ (an extemporaneous mixture of neutral liposome, MPL from Salmonella enterica serotype Minnesota Re 595, and QS21 saponin in PBS, pH 7, Vaccine Formulation Laboratory, University of Lausanne) and sera were harvested 3 weeks after each vaccination. Under these conditions, LMQ induced a significantly higher antibody response than either of the two other adjuvants (Fig. 5E).

### Vaccination with IMX313 fusion constructs leads to increased germinal centre responses

The difference in immunogenicity and TRA between the protein-nanoparticle and soluble protein, and between the vaccine platforms could be due to the magnitude of GCs involved in priming the antibody response. Strong and durable antibody responses elicited by Pfs25-IMX313 in both viral-vector and protein-in-adjuvant vaccinations may suggest IMX313 multimerized antigen can efficiently induce GC formation in secondary lymphoid organs, where host B cells proliferate after encountering antigens and subsequently undergo isotype-switching as well as somatic hypermutation resulting in mature plasma cells and memory B cells^31^. We investigated the GC B cell response in both the inguinal draining lymph nodes (dLNs) and spleen after immunization with viral vector (ChAd63 only) and protein-in-adjuvant vaccines.

**Figure 6:**
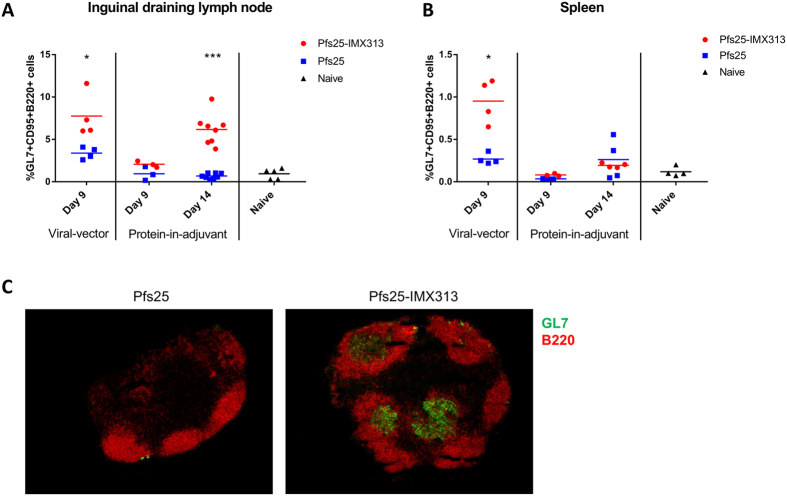
Germinal centre response after prime vaccination. BALB/c mice were immunised i.m. either with 1X10^8^ i.u. ChAd63 or 2.5 μg protein formulated in Alhydrogel for Pfs25 and Pfs25-IMX313. Nine days and 14 days post-vaccination the inguinal dLNs (**A**) and spleen (**B**) were harvested and surface stained as outlined in the methods section. The mean and individual % of GL7+CD95+ cells in the B220+ cell population are shown (gating strategy shown in Figure S3). On day 14 after protein-in-adjuvant immunisation, the inguinal dLNs were also sectioned and stained with fluorescence labelled anti-GL7 (green) and anti-B220 (red) antibodies. Representative sections from each group are shown (**C**). Mann-Whitney test was performed *p < 0.05, ***p < 0.001.

At day 9 post-vaccination (our unpublished work has shown this to be the peak of the GC response in both inguinal dLN and spleen after i.m. adenoviral immunization expressing ovalbumin) with 1 × 10^8^ i.u. ChAd63 Pfs25-IMX313 and ChAd63 Pfs25, the dLNs were harvested, cells were isolated and stained for the GC-markers GL7, CD95 and B220. The gating strategy used to identify GC B cells is defined in Figure S3. The % of GL7+CD95+ cells in B220+ cell population in the dLNs was significantly higher in the ChAd63 Pfs25-IMX313 vaccinated group, than the ChAd63 Pfs25 group (Fig. 6A). A similar effect was also seen in the spleen (Fig. 6B).

At day 9 post 2.5 μg protein-in-adjuvant (Alhydrogel) immunization, the % GC B cells in the inguinal dLNs were not significantly different between the Pfs25-IMX313 protein-nanoparticle and monomeric Pfs25 vaccinated groups (Fig. 6A). At day 14 however, the Pfs25-IMX313 nanoparticle induced significantly higher GC B cells than monomeric Pfs25 (Fig. 6A). Interestingly, only a minor increase in GC B cells was detected in the spleen on day 14 and there was no significant difference between soluble Pfs25 and Pfs25-IMX313 protein-nanoparticle vaccinated groups (Fig. 6B). Together, these results demonstrate Pfs25-IMX313 in both adenovirus and protein-in-adjuvant regimes is able to induce a stronger GC B cell response.

To confirm the difference in GC response observed at day 14 in the dLNs of mice immunized with protein-in-adjuvant vaccines (Fig. 6B), immunohistological studies were performed. Sectioned dLN samples were stained with anti-GL7 (to identify GCs) and anti-B220 (to identify B cell follicles) antibodies conjugated to AlexaFluor-488 and Phycoerythrin respectively. Stained dLNs were then visualised by fluorescence microscopy (DMI3000B, Leica Microsystems, UK). GCs (GL7+ B220+ double positive cells) were clearly stained in dLN sections from the Pfs25-IMX313 protein-nanoparticle vaccinated mice whilst there was no staining observed in the mice vaccinated with monomeric Pfs25 (Fig. 6C).

## Discussion

Anti-Pfs25 antibodies induced in humans by vaccination are able to block the sexual development of the parasite in the mosquito but it is critical that high antibody titers are induced in order to obtain significant transmission-blocking efficacy^8^. In this study we have fused Pfs25 to IMX313, a oligomerization technology, leading to the expression of a nanoparticle either from viral vectors (ChAd63 and MVA) or as a secreted product in *P. pastoris*. In both cases Pfs25-IMX313 shows about 10-fold greater antibody immunogenicity and significantly better transmission-blocking efficacy in membrane feeding assays than Pfs25 alone. Notably, when mice were vaccinated with different doses (2.5 μg, 5 μg and 10 μg) of Pfs25-IMX313 formulated in Alhydrogel, a clear “dose-sparing” effect was observed suggesting that a lower vaccination dose could achieve significant transmission-blocking efficiency. A long-term memory study of 250 days revealed that the antibody responses were maintained over time which is essential for a TBV. The fusion of Pfs25 to IMX313 does not affect the binding of vaccine-induced anti-Pfs25 to native protein antigen on the surface of *P. berghei* ookinetes transgenic for Pfs25.

The anti-Pfs25 IgG was tested for functional activity in the SMFA and the total IgG from the Pfs25-IMX313 vaccinated groups exhibited stronger TRA largely due to the higher levels of antigen-specific IgG as measured by the standardized ELISA. In addition to the difference in the antibody quantity, there was also a significant difference in the quality of antibody in the case of the protein-in-adjuvant vaccine, calculated from the SMFA data (Fig. 4D) but this difference was not seen for the viral vectored vaccine immunized groups. The presentation of the antigen might be different when it is multimerized by fusion to IMX313 which could account for the difference in quality of the antibody response. The role of IgG avidity in functional activity in the SMFA remains poorly understood but the avidity of the antibodies were also significantly higher in the Pfs25-IMX313 protein-nanoparticle group than in the monomeric Pfs25 group. The NaSCN-displacement ELISA employed here represents a relatively crude measure of the overall avidity of the polyclonal antigen-specific IgG response. We did not observe a difference in the avidity of the antibodies induced after vaccination with viral vectors. Notably, the quality differences of antibodies measured by SMFA and by avidity ELISA are matched (i.e., there are significant differences between Pfs25 and Pfs25-IMX313 when using the protein-in-adjuvant regime, but not with the viral vector regime). Using IgG1 and IgG2a as markers for Th2- and Th1-type response respectively, we also assessed the IgG isotype profile of the antibodies generated after vaccination with Pfs25 and Pfs25-IMX313 constructs using both viral-vector and protein-in-adjuvant regimes. ChAd63-MVA expressing Pfs25 induced a balanced IgG1 and IgG2a responses for both the monomeric and multimeric antigens. For the protein-in-adjuvant vaccinations, the soluble Pfs25 protein administered in Alhydrogel predominantly induced IgG1 consistent with the understanding of this Alum-based adjuvant being a weak Th1-type response inducer^26^. Interestingly, when Pfs25 was fused to IMX313 and administered in the same adjuvant, a significant increase in IgG2a was measured suggesting that the particulate nature of the antigen polarized the immune responses to a Th1-type response. This result is consistent with previous observation that vaccination using viral vectors expressing antigen fused to IMX313 (adenoviral^32^ and MVA^16^) significantly improved IFN-γ secreting T helper cell (Th1) responses.

Multimerization of Pfs25 in both the vaccine platforms also induced significantly higher GC B cells than monomeric Pfs25. The significant difference in the induction of GCs probably resulted in the corresponding difference in their antibody responses. Previous studies have shown that combining viral vectored and soluble protein-in-adjuvant vaccines can generate an immune response which equals, or in some cases surpasses, the best immune response observed for either viral vectors or soluble protein alone^33^. In our study, we compared the ability of different vaccination regimes to induce the highest anti-Pfs25 antibody titers and the 2 dose protein-in-adjuvant regime was found to be the best for Pfs25-IMX313 nanoparticle when administered in Matrix M. In addition, the immunogenicity of this protein-nanoparticle can be further enhanced by using more potent adjuvants especially with the preclinical adjuvant LMQ. Finally, as an important consideration in determining whether the IMX313 multimerization technology is suitable for human use, we measured whether immunization with IMX313 fusion proteins induced antibodies capable of recognising HC4bp, despite the low (21%) similarity of the primary sequences. Our work showed that antibodies generated by immunization with the Pfs25-IMX313 nanoparticle did not recognise HC4bp by ELISA. This result is unsurprising since it has previously been shown (Table 3 in ref. 15) that antibodies raised against murine C4bp did not recognise human C4bp; as shown in the alignment, murine C4bp shares 26 identical residues in an overlap of 54 amino acids with human C4bp (48%), whereas IMX313 shares 11 identical residues in an overlap of 52 amino acids (21%) (Fig. S1A).

Taken all together, our study demonstrates that multimerization of antigen by fusion with IMX313 is a highly promising vaccine delivery technology for Pfs25. This finding supports previous observations that particle-like structures improve the immunogenicity of recombinant malaria antigens such as *P.falciparum* circumsporozoite protein (CSP) carried by hepatitis B surface antigen (HBsAg) VLP^34^, *P.vivax* CSP carried by HBsAg VLP^35^ or multilamellar vesicles^36^; and Pfs25-EPA^11^, Pfs25-OMPC^12^ and Pfs25-CP VLP^13^. The production and purification of this secreted nanoparticle is simple and cost effective. The antibodies are functional and, because high anti-Pfs25 antibody titers are crucial for significant transmission blocking activity in humans, the immune-potentiating effect of multimerization of Pfs25 with IMX313 may be an efficient and effective platform to achieve these titers. A Phase Ia clinical trial of the viral vectors (ChAd63 and MVA) expressing Pfs25-IMX313 is planned to start in 2015 as part of an EU FP7-funded programme (MultiMalVax, www.multimalvax.eu) for the development of multi-stage malaria vaccines.

## Materials and Methods

### Generation of viral-vectored vaccines expressing Pfs25-IMX313

Pfs25 sequence (GenBank accession no: AAN35500, from Alanine-22 to Threonine-193) with four potential N-linked glycosylation sites (112, 165, 187 and 202) mutated (as described in^37^), was codon optimized for expression in humans (GeneArt® Life Technologies, Germany). The predicted native signal peptide was replaced with the human tissue plasminogen activator (tPA) signal peptide sequence (GenBank Accession No. K03021) as described before^38^. For the Pfs25-IMX313 constructs a 229 bp DNA fragment encoding the IMX313 domain was cloned at the C-terminus of Pfs25. The Pfs25 and Pfs25-IMX313 inserts were subcloned into the ChAd63 and MVA destination and shuttle vectors. The recombinant viral vaccines were generated as described previously^18^,^39^.

### Generation of Pfs25 and Pfs25-IMX313 in *P. pastoris*

Pfs25 and Pfs25-IMX313 sequences (as above) were cloned with a C-terminal His6-tag (Pfs25) or an N-terminal hexahistidine-tag (Pfs25-IMX313) into the PichiaPink (Invitrogen^TM^ Life Technologies, UK) protein expression plasmid (pPinkα-HC) containing the *Saccharomyces cerevisae* α-mating factor pre-sequence for secretion of the protein. 5 μg to 10 μg of linearized plasmid DNA was electroporated (Bio-Rad, UK) into the PichiaPink strain of *P. pastoris* according to the protocol provided by Invitrogen (TM). Transformed colonies were screened by small-scale (5 mL) protein expression followed by western blot analysis to identify the transformed clone which expressed and secreted the highest level of protein. Large-scale (2 L) protein expression was performed according to Invitrogen’s PichiaPink protocol. After protein expression, the culture supernatants were filtered through a Steritop™ bottle top filter unit with 0.22 μm PES Membrane (Merck Millipore, UK). The sample was immediately loaded onto a HisTrap excel column (GE healthcare, UK) using a peristaltic pump (Gilson, UK). The column was washed with 5 column volumes (CV) of PBS containing 10 mM imidazole (pH 7.4) and the protein was eluted into fractions using 500 mM imidazole in PBS (pH 7.4). Fractions with protein were then further purified by size exclusion chromatography using an AKTA purifier (GE healthcare, UK) with a Superdex 200 pg gel filtration column (GE healthcare, UK). Purified proteins were concentrated using Amicon® Ultra centrifugal filter units (Merck Millipore, UK) and the protein concentration was determined by nanodrop (Thermo Scienctific) and confirmed by Pierce^TM^ bicinchoninic acid (BCA) protein assay (Thermo Scientific, UK).

### Western Blot Analysis

To determine the expression of the recombinant antigens expressed by viral-vectors in mammalian cells, 1 × 10^7^ cells/mL HEK293 cells were seeded onto 6 well plates and transfected with pENTR4-LPTOS shuttle plasmid DNA (expressing Pfs25 and Pfs25-IMX313) using Lipofectamine™ 2000 (Invitrogen, UK). Cells were incubated for 48 hours at 37 °C and 5% CO2. Supernatant were harvested for western blot analysis. Western blots were performed using standard methods^40^. Briefly, after polyacrylamide gel electrophoresis and transfer to blotting membrane, blots were incubated with the monoclonal primary antibody 4B7 for 1 h and washed with PBS in 0.05% Tween 20 (PBS/T) for 30 min. After washing, the blots were incubated with alkaline phosphatase conjugated donkey-anti-mouse IgG secondary antibodies (Jackson Immuno Research, USA) for 1 h, and washed in PBS/T for 30 min. Blots were then rinsed briefly in deionized water and incubated with 1 SIGMAFAST™ BCIP®/NBT tablet (Sigma-Aldrich, UK) dissolved in 10 ml dH_2_O until the desired level of staining was achieved.

### Vaccinations

All animal experiments and procedures were performed according to the UK Animals (Scientific Procedures) Act Project Licence (PPL 30/2414 and 30/2889) and approved by the Oxford University Local Ethical Review Body. Age-matched female BALB/c mice (Harlan, UK), housed in specific-pathogen free environments, were vaccinated with equal amount of vaccines into each legs via the intramuscular route (i.m.) using either a heterologous prime-boost viral-vectored regime or protein-in-adjuvant prime-boost regime. For the viral-vector vaccinations, mice were primed with 1 × 10^8^ i.u. ChAd63 and boosted 8 weeks later with 1 × 10^7^ pfu MVA. Control vaccinations were performed with ChAd63 and MVA expressing green fluorescent protein (GFP). Vaccines were prepared in sterile endotoxin-free PBS (Sigma-Aldrich, UK). For the protein-in-adjuvant immunizations, the proteins were formulated with the adjuvant prior to vaccination as follows: Alhydrogel (85 μg Alhydrogel per dose was mixed with antigen in 20mM Tris buffer and incubated at room temperature (RT) for 1 h before injection); Matrix M (12 μg of Matrix M per dose in mice mixed with antigen in PBS); MF59 (mixed with antigen in PBS at a 1:1 volume ratio and 25 μL of MF59 per dose in mice); LMQ (30 μg of LMQ per dose in mice mixed with antigen in PBS).

### Immunofluorescence Assay

The recognition of native parasite Pfs25 antigen by the vaccine-induced antiserum was confirmed via immunofluorescence assay on paraformaldehyde (PFA) fixed ookinetes. This method has been described previously^18^. Briefly, Pfs25DR3 transgenic *P. berghei* parasite (provided by Dr Andrew Blagborough, Imperial College, London) ookinete culture smears were fixed for 10 min in 4% PFA/PBS followed by washing in PBS. Slides were blocked for 1 h in blocking buffer (10% v/v goat serum, 1% w/v BSA in PBS) followed by overnight incubation with 4B7 mAb (1:500) or test antiserum (1:1000) in 1% (w/v) BSA in PBS at 4 °C in a wet chamber. The following day, slides were washed three times in PBS and then incubated with Alexa Fluor 488-conjugated goat anti-mouse IgG (1:2000) for 60 min. After another three washes, slides were mounted with mounting medium with DAPI (Vector Laboratories, UK) and analyzed by fluorescence microscopy on a DMI3000B microscope (Leica Microsystems, UK).

### Pfs25 standardized ELISA, Avidity ELISA and Endpoint ELISA

ELISA against Pfs25 was performed according to a standardized protocol using a reference serum. This protocol has been described previously^25^. Briefly, Nunc-Immuno maxisorp plates (Thermo Scientific, UK) were coated with *P. pastoris* expressed monomeric Pfs25 protein at 0.1 μg per well at RT. Plates were washed with PBS/T and blocked for 1 h with 5% skimmed milk in PBS. Test serum samples were diluted and added followed by incubation for 2 h at RT and then washed with PBS/T as before. Donkey anti-mouse total IgG conjugated to alkaline phosphatase (Jackson ImmunoResearch Laboratories, USA) was added to the plate (1:5000 dilution in PBS) for 1 h at RT. After a final wash in PBS/T, p-nitrophenylphosphate (Sigma-Aldrich, UK) diluted in diethanolamine buffer (Thermal Scientific, UK) was used as a developing substrate. Optical density (OD) was read at 405 nm using an ELx800 absorbance microplate reader (Biotek, UK). All samples were tested against a serially diluted standard reference serum (supplied by NIH as was previously reported^12^) with a known antibody titer. The absorbance of individual test samples was converted into arbitrary antibody units (AU) using a standard curve generated by this standard reference serum.

Antibody avidity was assessed using a sodium thiocyanate (NaSCN)-displacement ELISA. Nunc-Immuno maxisorp plates were coated with recombinant Pfs25 protein over-night, blocked and then washed with PBS/T as before. All individual serum samples were diluted so that each sample contained the same level of Pfs25 AU (in this study 10 AU). Samples were added in duplicate, following incubation and washing, an ascending concentration of the NaSCN (0–7M) was added to the wells. The plates were incubated at RT for 15 min followed by washing and further development as before. The avidity ELISA readout was the intercept of the curve (molar concentration of NaSCN/OD) where OD reached a 50% reduction of the OD in NaSCN-free samples.

For IMX313 and HC4bp endpoint ELISA, Nunc-Immuno maxisorp plates were coated with the corresponding proteins as before. Serum samples were added in duplicates and diluted 3 fold down the plate, followed by the same procedure as for the Pfs25 standardized ELISA. The endpoint titer is defined as the X-axis intercept of the dilution curve at an absorbance value (±three standard deviations) greater than the OD for a serum sample from a naïve mouse. For the IgG subclass endpoint ELISA, biotinylated anti-mouse IgG1 or IgG2a (BD bioscience) was used as the secondary antibodies. After incubation and wash, alkaline phosphatase conjugated ExtrAvidin (Sigma-Aldrich, UK) was added at 1:5000 in PBS and incubated for 30 min at RT. The development and measurement of OD was done as before.

### IgG Purification

Total IgG was purified from day 70 (after viral vector immunization) or day 62 (after protein-in-adjuvant immunization) pooled mouse sera (300 μL serum from all mice in a group was pooled irrespective of individual antibody titers). IgG was also purified for the vector-control and OVA immunized mice as negative control. The IgG was purified using Protein G columns (Pierce, USA). Briefly, Protein G columns were equilibrated with binding buffer (Immunopure IgG, Pierce, USA) after which a 1:1 mixture of sera and binding buffer was allowed to flow through under gravity, washed and eluted with elution buffer (Immunopure IgG, Pierce, USA). The eluted fraction was collected in 1 M Tris-HCl (pH 9.0, Teknova, USA) and transferred for buffer exchange to Amicon centrifugal filters (Millipore, USA) using PBS. The eluent was concentrated in PBS (Invitrogen, UK) and filtered using a 0.22 μm Millipore Ultrafree sterile centrifugal unit. Total protein was quantified using a NanoDrop spectrophotometer at 280 nm and adjusted to 4 μg/ml.

### SMFA

The ability of vaccine-induced antibodies to block the development of *P. falciparum* strain NF54 was evaluated by SMFA as previously described^41^. The percentage of mature Stage V gametocytes was adjusted to 0.15% ± 0.05% and the male-female ratio is stable (almost always 1 male: 2–3 female). These were mixed with purified IgG at the concentrations (diluted in PBS) shown in the figures and then fed to 4–6 day old starved female *A. stephensi* (SDA 500) via a parafilm^®^ membrane. The mosquitoes were maintained at 26 °C and 80% relative humidity. After 7 days, midguts from twenty mosquitoes per group were dissected, oocysts counted and the number of infected mosquitoes recorded. Percent reduction in infection intensity was calculated relative to the respective control IgG tested in the same assay.

### Germinal centre staining

Inguinal dLNs and spleens were prepared as described^42^. Cells were transferred to 96 well V-bottom plates (Thermo Scientific, UK) and centrifuged (300 × *g*, 5 min). Supernatants were then discarded and cells were resuspended with addition of Fc Receptor Block (1:50) (eBioscience, UK) for 15 min. Following a wash in FACS buffer, cells were stained with anti-GL7 AlexaFluor 647 (eBioscience, UK) at 1:100 dilution, anti-CD95 PE (eBioscience, UK) at 1:100 dilution and anti-B220 PeCy7 (eBioscience, UK) at 1:200 for 30 min at RT in the dark. After this incubation, cells were washed twice and resuspended in FACS buffer and data were acquired on a LSRII (BD Bioscience, UK) and analyzed by FlowJo (TreeStar Inc, USA).

### Lymph node sectioning and germinal centre staining

Mice were sacrificed at day 14 after i.m. immunization. The inguinal dLN were excised and immediately frozen in Optimal Cutting Temperature compound (Thermo Scientific, UK) on dry ice. The sectioning was performed using a Cryostat Leica CM3050S (Leica Biosystems, UK). 10 μm sections were taken and fixed on glass slides. Slides were stored in −80 °C before immunostaining.

Prior to staining the slides containing the dLN, sections were fixed in 100% acetone for 10 min at −20 °C and then air dried for 30 min. Slides were rehydrated in PBS for 10 min and then blocked with 5% PBS/BSA for 45 min. After a wash with PBS, the slides were incubated with AlexaFluor-488 conjugated anti-mouse GL7 (eBioscience, UK) (1:100) and PE conjugated anti-mouse B220 (eBioscience, UK) (1:100) antibodies at RT for 1 h. The slides were then washed 3 times for 10 min each with PBS. After this, FLuoromount G mounting medium (eBioscience, UK) was applied to the slides and coverslips were placed. Slides were then visualized using DMI3000B (Leica Microsystems, UK). Digital images of the slides were taken and edited by ImageJ (NIH, USA).

### Statistical analysis

Continuous variables between two groups were compared by a Mann-Whitney test, and those among three or more groups were compared by a Kruskal-Wallis test. If significant, a Dunn’s multiple comparison post-test was performed. To evaluate a dose-effect (2.5, 5 or 10 μg/dose) in antibody responses measured by ELISA at days 20, 41 and 62, a linear regression analysis was performed using Log_10_ transformed antibody responses for each vaccine group (Pfs25 or Pfs25-IMX313). The dose and day factors were included as continuous variables in the model.

A difference in quality of anti-Pfs25 antibodies judged by SMFA was evaluated using a linear regression model. The log_10_ transformed ratio of the mean oocyst count in control and test samples was the dependent variable, and the square root of anti-Pfs25 antibody level (measured by ELISA) and vaccine group (Pfs25 or Pfs25-IMX313) were dependent variables in the model. Holm’s adjusted p-values are shown. Since log of mean ratio became infinity when a test IgG had zero oocysts on average, such data were excluded from the analysis (ChAd63-MVA Pfs25-IMX313 IgG tested at 750 μg/ml of total IgG, and protein-in-adjuvant Pfs25-IMX313 IgGs tested at 375 and 750 μg/ml in one of feeding experiments). All statistical tests were performed in Prism 6 (GraphPad Software Inc, USA), or JMP11 (SAS Institute Inc, USA) and p-values <0.05 were considered significant.

## Additional Information

**How to cite this article**: Li, Y. *et al.* Enhancing immunogenicity and transmission-blocking activity of malaria vaccines by fusing Pfs25 to IMX313 multimerization technology. *Sci. Rep.* **6**, 18848; doi: 10.1038/srep18848 (2016).

## Supplementary Material

Supplementary Information
