# Supplementary material for: Enhancing immunogenicity and transmission-blocking activity of malaria vaccines by fusing Pfs25 to IMX313 multimerization technology
Source: Sci Rep. 2016 Jan 8;6:18848. doi: 10.1038/srep18848 (PMC4705524; doi:10.1038/srep18848)
Supplement: Supplementary Information [file srep18848-s1.pdf]

Title

Enhancing immunogenicity and transmission-blocking activity of malaria vaccines by fusing Pfs25 to IMX313 multimerization technology.

Authors

Yuanyuan Li[1], Darren B. Leneghan[1*], Kazutoyo Miura[2], Daria Nikolaeva[1,2], Iona J. Brian[1], Matthew D. J. Dicks[1], Alex J. Fyfe[1], Sarah E. Zakutansky[1], Simone de Cassan[1], Carole A. Long[2], Simon J. Draper[1], Adrian V. S. Hill[1], Fergal Hill[3], and Sumi Biswas[1*]

[1]Jenner Institute, University of Oxford, Oxford, OX3 7DQ, UK.

[2]Laboratory of Malaria and Vector Research, National Institute of Allergy and Infectious. Disease / National Institutes of Health, Rockville, Maryland, USA.

[3]IMAXIO, 69007 Lyon, France.

*To whom correspondence should be addressed:

lee.li@ndm.ox.ac.uk , darren.leneghan@ndm.ox.ac.uk , sumi.biswas@ndm.ox.ac.uk.

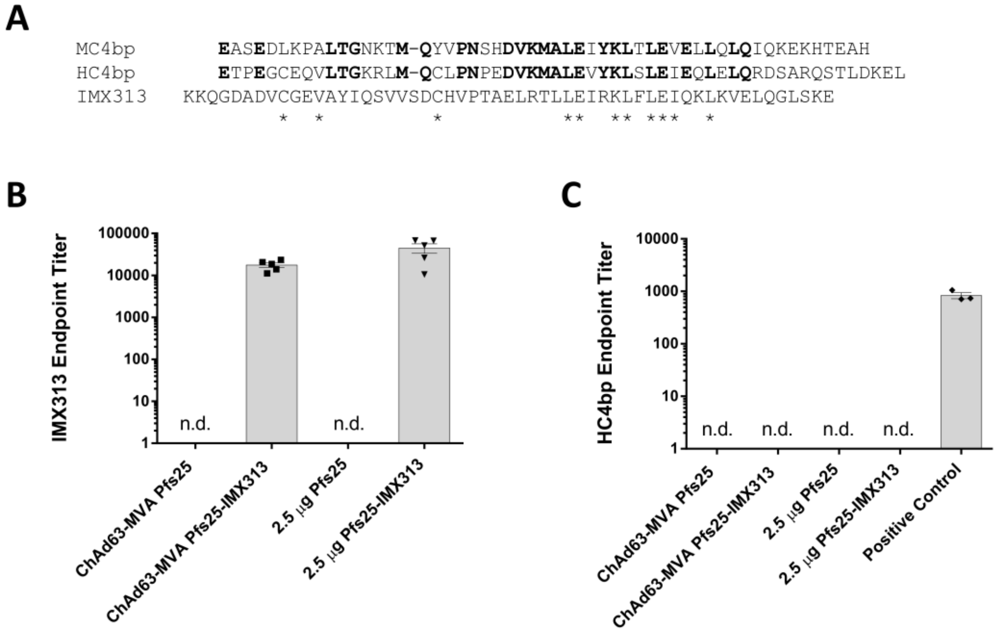

Figure S1. Vaccination with Pfs25-IMX313 does not induce antibodies reactive against HC4bp.

(A) The peptide sequences of murine C4bp (MC4bp), human C4bp (HC4bp) and IMX313 are aligned. The identical peptide sequence matches between murine and human are bolded and sequence matches between human and IMX313 are marked with *.

(B and C) Day 70 and 62 sera from mice previously vaccinated with Pfs25 and Pfs25-IMX313 (Fig. 2B and 2C, 2.5µg group) were tested for their antibody response against IMX313 protein (B) and HC4bp (C). Sera from mice (n=3) received HC4bp vaccination were used as positive control for HC4bp ELISA. Mean with SEM are depicted and individual data are shown for B and C (n.d., not detected).

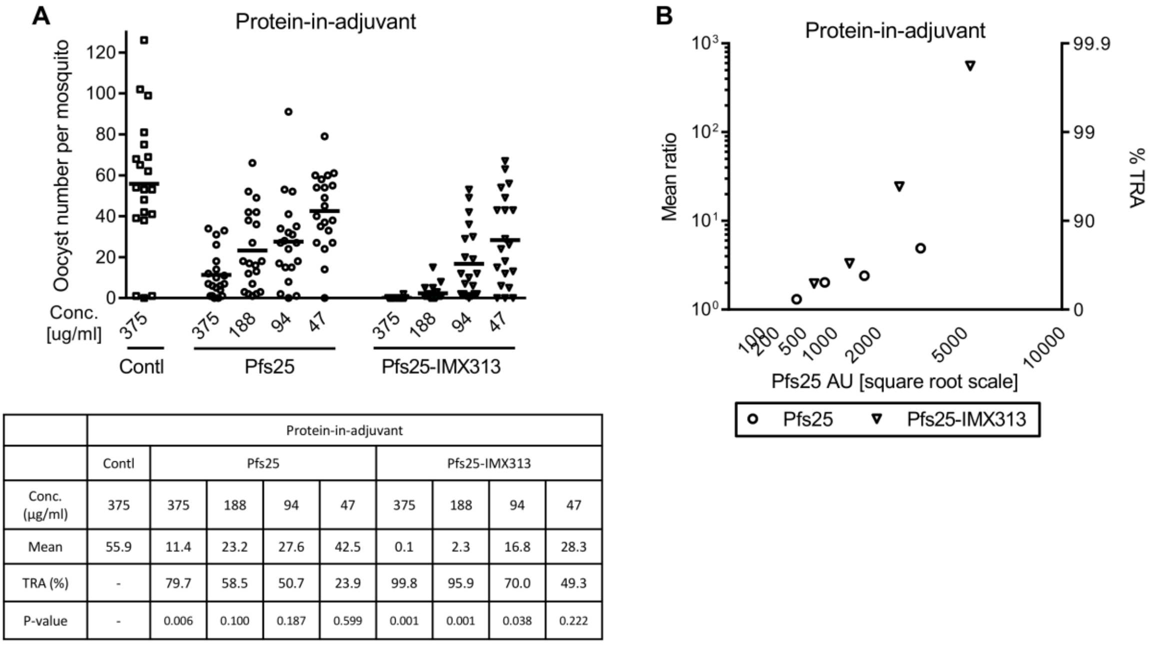

	Protein-in-adjuvant								
	Contl	Pfs25				Pfs25-IMX313			
Conc. (µg/ml)	375	375	188	94	47	375	188	94	47
Mean	55.9	11.4	23.2	27.6	42.5	0.1	2.3	16.8	28.3
TRA (%)	-	79.7	58.5	50.7	23.9	99.8	95.9	70.0	49.3
P-value	-	0.006	0.100	0.187	0.599	0.001	0.001	0.038	0.222

Figure S2. Additional assessment on effect of mouse IgG induced by protein-in-adjuvant vaccination on *P. falciparum* NF54 parasite infectivity in *A. stephensi* mosquitoes.

The same total purified IgGs from day 62 pooled serum of mice that received 3 doses of 2.5 µg Pfs25 or Pfs25-IMX313 formulated in Alhydrogel were tested again in an independent feeding assay (replication of Fig. 4). Data points represent the number of oocysts in individual mosquitoes and the lines show the arithmetic mean (A). The table shows the mean number of oocysts per mosquito and percentage of transmission reducing activity with p-value. The square root of anti-Pfs25 specific IgG level in the feeder is shown on the x-axis. The ratio of mean oocyst counts in control and test samples is plotted on a log scale along the left y-axis, and the associated % TRA is plotted along the right y-axis (B).

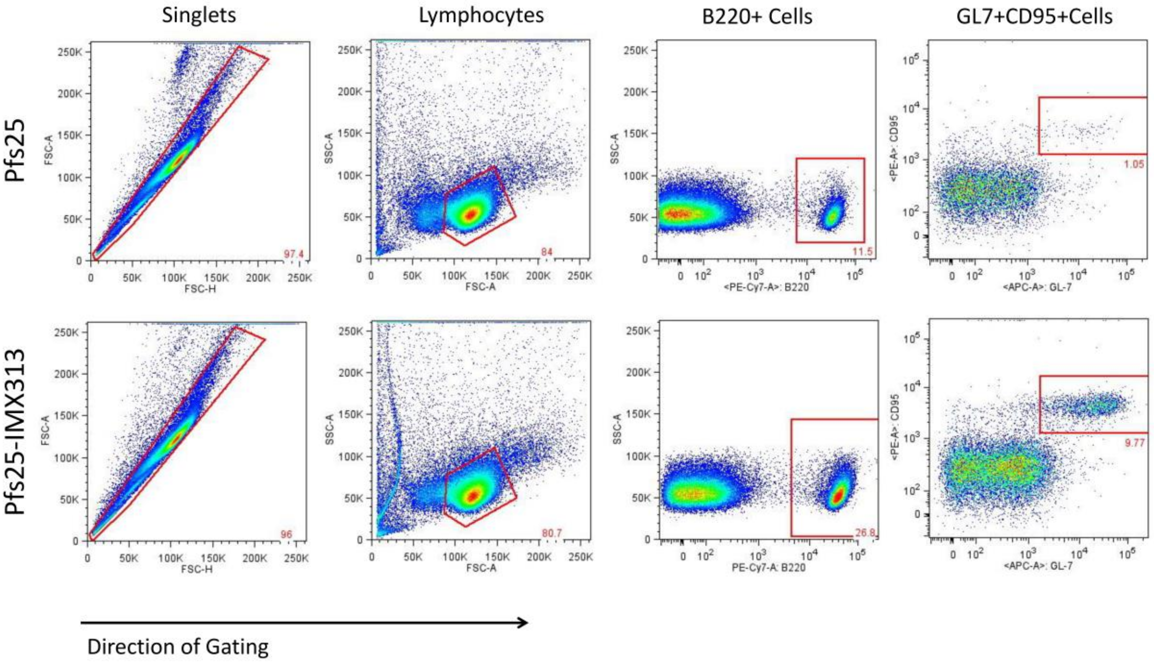

Figure S3. Gating strategy to identify germinal centre B cells.

BALB/c mice (n=5 / group) were immunised i.m. with 1X10^{8} i.u. ChAd63 or 2.5 µg protein formulated in Alhydrogel. Nine or 14 days later, inguinal dLN and spleen were harvested and surface stained for GC B cells (GL7+CD95+B220+). The sequential gating strategy for GC B cells is shown and the gates show the percentage of parent.
